# A Novel Mutation in β Integrin Reveals an Integrin-Mediated Interaction between the Extracellular Matrix and *cki-1/p27^KIP1^*

**DOI:** 10.1371/journal.pone.0042425

**Published:** 2012-08-06

**Authors:** Shingo Kihira, Eun Jeong Yu, Jessica Cunningham, Erin J. Cram, Myeongwoo Lee

**Affiliations:** 1 Department of Biology, Baylor University, Waco, Texas, United States of America; 2 Department of Biology, Northeastern University, Boston, Massachusetts, United States of America; Virginia Commonwealth University Medical Center, United States of America

## Abstract

The cell-extracellular matrix (ECM) interaction plays an essential role in maintaining tissue shapes and regulates cell behaviors such as cell adhesion, differentiation and proliferation. The mechanism by which the ECM influences the cell cycle *in vivo* is poorly understood. Here we demonstrate that the β integrin PAT-3 regulates the localization and expression of CKI-1, a *C. elegans* homologue of the cyclin dependent kinase inhibitor p27^KIP1^. In nematodes expressing wild type PAT-3, CKI-1::GFP localizes primarily to nucleoli in hypodermal cells, whereas in animals expressing mutant *pat-3* with a defective splice junction, CKI-1::GFP appears clumped and disorganized in nucleoplasm. RNAi analysis links cell adhesion genes to the regulation of CKI-1. RNAi of *unc-52*/perlecan, *ina-1/α integrin*, *pat-4*/ILK, and *unc-97*/PINCH resulted in abnormal CKI-1::GFP localization. Additional RNAi experiments revealed that the SCF E3 ubiquitin-ligase complex genes, *skpt-1*/SKP2, *cul-1*/CUL1 and *lin-23*/F-box, are required for the proper localization and expression of CKI-1, suggesting that integrin signaling and SCF E3 ligase work together to regulate the cellular distribution of CKI-1. These data also suggest that integrin plays a major role in maintaining proper CKI-1/p27^KIP1^ levels in the cell. Perturbed integrin signaling may lead to the inhibition of SCF ligase activity, mislocalization and elevation of CKI-1/p27^KIP1^. These results suggest that adhesion signaling is crucial for cell cycle regulation *in vivo*.

## Introduction

Integrins are αβ heterodimeric receptors that mediate bi-directional interactions between cells and extracelluar matrix (ECM) [1]. In mammals, 13α and 8β chains comprise more than twenty heterodimers and play important roles in controlling cell behaviors such as cell adhesion, migration and proliferation [2]. Among the β subunits, β1 integrin is broadly expressed and has multiple splice variants. For example, four β1 splice variants, β1A, β1B, β1C and β1D differing in their cytoplasmic tails, are expressed in many tissues [3]. β1A is the dominant splice form and is expressed ubiquitously [3]. β1D is produced by alternative splicing and is found in striated muscle cells only [4]. Both β1A and β1D forms are localized to focal adhesions and retain the conserved NPxY phosphorylation motif [5], [6]. However, the β1B variant, expressed in keratinocytes and hepatocytes, fails to localize to focal adhesions and exhibits dominant negative activity to β1A-paired integrins [7], [8]. β1B is the result of mis-splicing of intron 7, and retains intronic sequence in its mRNA [9]. β1C is expressed in normal tissues, such as the prostatic epithelium, and is downregulated in cancer cells [10]. β1C integrin is produced from an alternative splicing event in the cytoplasmic tail of β1 integrin, usually includes exon C and results in a protein 27 amino acids longer than the regular β1A splice form [5], [11].

In many cancerous conditions, integrins lose their connection to the ECM or change their expression patterns [3], [12], [13]. The ECM also undergoes remodeling, resulting in abnormal deposition of proteins or increased ECM stiffness. A change in ECM composition or mechanical properties may upregulate integrin signaling, which promotes cell survival, adhesion and proliferation [14], [15]. For example, cell detachment from the ECM increases the level of cyclin dependent kinase (CDK) inhibitors thereby preventing advancement to S phase of the cell cycle [16].

In some cases, integrin signaling can promote cell cycle arrest [17]. For example, the expression of integrin β1C in mammalian cells increases the level of p27^KIP1^, a CDK inhibitor [18]. In contrast, lowering the level of p27^KIP1^ allows activation of the CDK/cyclin complex and promotes the cell cycle transition from G1 to S [19]–[22]. In these cells, adhesion to the ECM activates an E3 ubiquitin ligase that is essential for the degradation of p27^KIP1^. The expression of SKP2, an important component of the SCF ubiquitin ligase (E3) complex, is also dependent on cell adhesion at the G1 to S transition [23]. However, there is little information available about how integrin signaling regulates the level of cell cycle inhibitors like p27^KIP1^ *in vivo*.

The nematode *Caenorhabditis elegans* expresses only two integrins, PAT-3/INA-1 [24] and PAT-3/PAT-2 [25], which simplifies the analysis of genetic interactions between integrin and cell cycle control genes. Overexpression of *C. elegans* p27^KIP1^/CKI-1 has been found to induce growth arrest and the *cki-1* null mutation results in hyperplasia of tissues such as the hypodermis, the vulva and the intestine [26]–[28]. The disruption of *cki-1* also results in the production of extra distal tip cells from the somatic gonad lineage [29], [30]. The activity of *cki-1* is regulated by the coordination of stage-specific cellular events such as binding to the CDK/cyclin complex and a sharp increase in expression at the late larval and young adult stages [26], [31]–[33].

This study the role of *pat-3(sp)* (previously known as *pat-3*(*β1C*)) builds on our previous work on the function of PAT-3 signaling in *C. elegans* [34]. *pat-3(sp)* is a frameshift mutation in the splice acceptor region (ag to aa) that abolishes conserved interaction domains such as the NPxY motifs and creates a splice variant with an extra 19 amino acids. The *pat-3(sp)* animals not only produce mutant *pat-3*, but also express the regular splice form due to utilization of an unusual splice acceptor [34]. *pat-3(sp)* has similarities to the human β1B and β1C integrins. The *pat-3(sp)* mutant is similar to β1B because it retains intron sequence in the mRNA and the transgenic line expresses mutant and normal splice forms simultaneously. The mutant is similar to the β1C variant in that it is expected to produce a longer PAT-3 lacking the NPxY motifs [34].

In this study, we assessed the role of *pat-3(sp)* in cell cycle regulation. Briefly, in transgenic *pat-3* rescued lines carrying *pat-3(sp)* [34], *cki-1::GFP* [26] was upregulated and exhibited a distinct sub-nuclear localization compared to wild type animals. RNA interference analyses revealed that the localization and level of CKI-1 are mediated by focal adhesion molecules and the SCF E3 ubiquitin ligase complex [35], [36]. Taken together, our findings suggest that integrin signaling, in conjunction with SCF E3 ligase complex activity, plays a crucial role in the localization and level of CKI-1 *in vivo*.

## Results

### βpat-3(sp) Increases CKI-1 Levels and Exhibits a Distinct Localization in Nuclei

**Figure 1:**
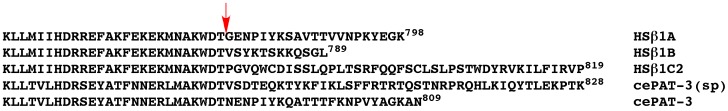
Sequences of the PAT-3 cytoplasmic tails. Wild type and mutant PAT-3 tails are compared to human β1A, β1B, and β1C cytoplasmic tails. Location of intron 7 is indicated by the red arrow.

PAT-3 β integrin is expressed in virtually all tissues in the nematode *C. elegans* [37], and is required for muscle development and function. Null mutations in *pat-3* cause a fully penetrant embryonic arrest due to defective muscle elongation [25], [38]. Previously, we created a mutation at the intron 7 splice junction in the cytoplasmic tail of PAT-3 integrin (Figure 1) [3], [34]. The transgenic rescued line, *pat-3(sp)*, is viable but exhibits cold-sensitive larval arrest with gonad and muscle defects. We found that *pat-3(sp)* expresses the non-spliced as well as the spliced *pat-3* mRNA, suggesting that mutant *pat-3* might inhibit the function of wild type *pat-3*. In this study, we have expanded our analysis to investigate the molecular function of *pat-3(sp)* [34].

**Figure 2:**
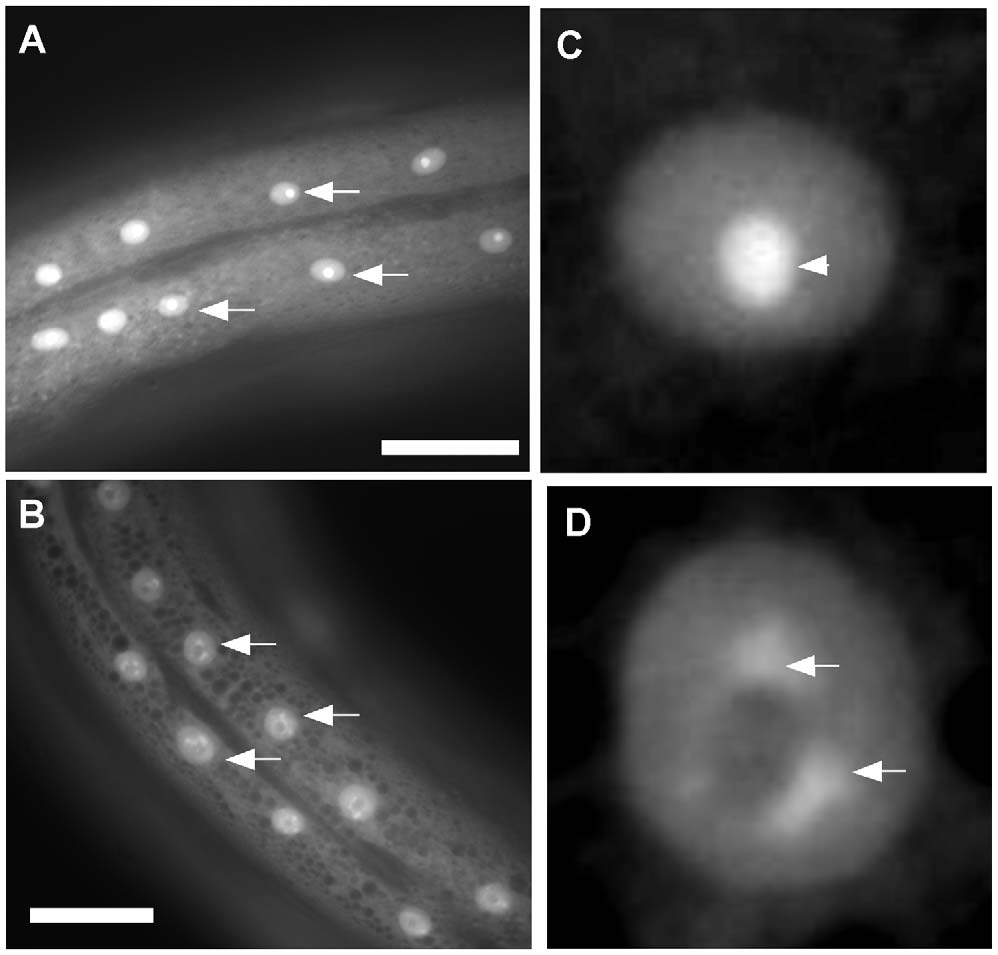
CKI-1::GFP is localized to the nucleus and nucleolus in *pat-3* transgenic rescued animals. CKI-1::GFP transgenic animals were examined using fluorescence microscopy. Panels A and B show mid-body regions of *pat-3(+)* and *pat-3(sp)* rescued animals, respectively. Arrows indicate the nuclei of hypodermal cells at early adult stages. Panels C and D depict a hypodermal nucleus in *pat-3*(+) and *pat-3(sp)* worms, respectively. CKI-1::GFP appeared to be nucleolar (arrow heads) in *pat-3(+),* while the CKI-1::GFP appeared clumped in *pat-3(sp)* nuclei (arrows). Scale bar = 50 µm.

Studies of the mammalian β1B and β1C integrins in mammalian cells revealed that expression of these integrins suppresses cell adhesion and proliferation [5] and upregulates the expression of p27^KIP1^ [39]. To investigate a potential linkage between PAT-3 and CKI-1, the *C. elegans* homolog of mammalian p27^KIP1^ [26], [29], [40], we created rescued transgenic lines containing *pat-3(+)* or *pat-3(sp)* genomic DNA by co-injecting with DNA encoding a CKI-1::GFP fusion protein [26], [34]. As previously described [26], [29], CKI-1::GFP is expressed in hypodermal cells of late L4 and young adult animals. Nuclear expression is observed within an ER meshwork, typical of the hypodermal syncytium (hyp 7) (Figure 2A and 2B) [41]. In *pat-3(+)* rescued animals, the appearance of CKI-1::GFP is a distinct fluorescent spot within a round green nucleus, suggesting nuclear and nucleolar localization (Figures 2A and 2C). To substantiate our interpretation that CKI-1::GFP localizes to the nucleolus, the *ncl-1* gene, disruption of which results in enlarged nucleoli, was depleted using RNAi [42], [43]. In the *pat-3(+)* background, *ncl-1* (RNAi) significantly increased the size of the CKI-1::GFP spot. ImageJ analysis showed that the size of the green spot was increased by 2.4 fold when compared to that of control RNAi animals (Figure S1, Table S1). Therefore, we conclude that the observed spots on the nuclei are likely to represent nucleolar localization.

**Figure 3:**
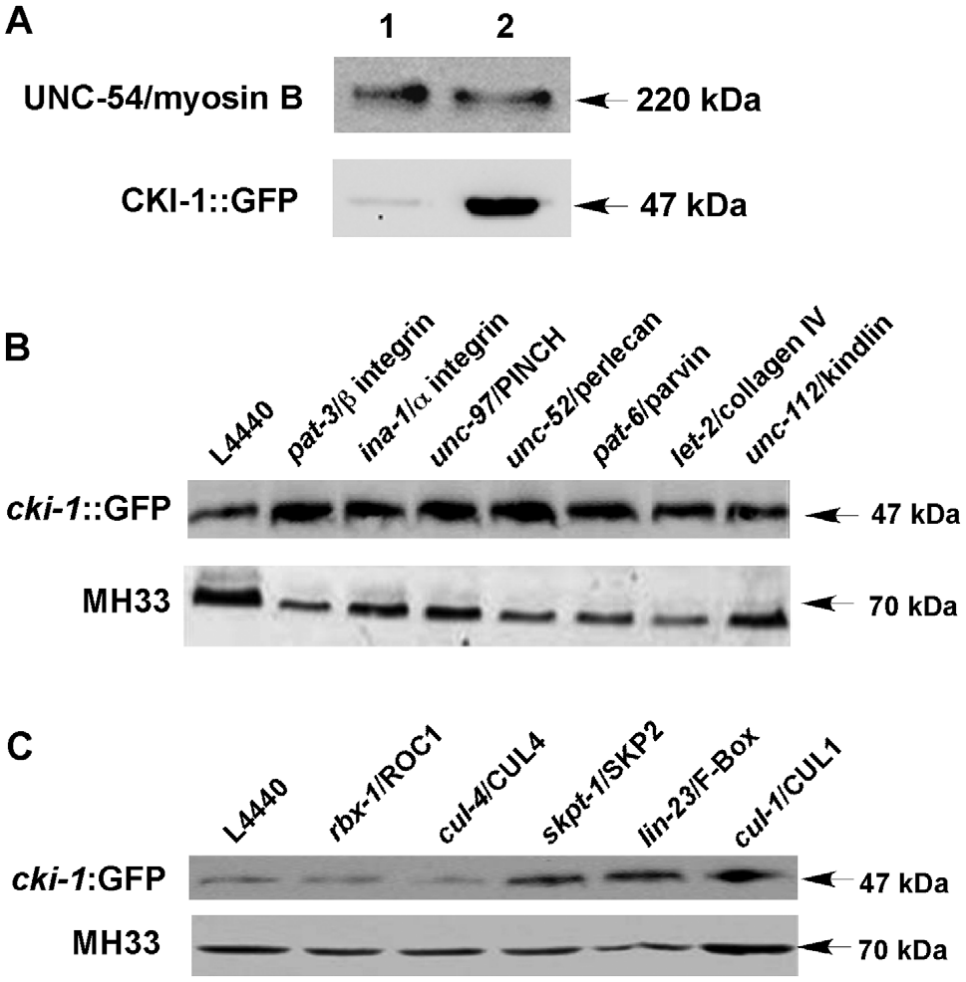
Immunoblot analysis of CKI-1::GFP protein in transgenic animals. Panel A: CKI-1::GFP expression levels were assessed in the transgenic rescued lines. Top bands in lanes 1 and 2 show the relative level of UNC-54/myosin B in each sample. Bottom bands indicate the level of CKI-1::GFP. Lanes 1 and 2 represent *pat-3*(+) and *pat-3(sp)*, respectively. Quantification (Table S1) using ImageJ software revealed that CKI-1::GFP level was 10-fold increased in *pat-3(sp)* animals compared to *pat-3*(+) animals. Panel B: CKI-1::GFP expression levels were assessed in *pat-3*(+) animals treated with RNAi directed against focal adhesion genes. Top bands represent the amount of CKI-1::GFP in extracts prepared from each RNAi condition. L4440 is a negative RNAi control. The *pat-3*, *ina-1*, *unc-97*, *unc-52, pat-6* and *let-2* RNAi caused upregulation of CKI-1::GFP, while *unc-112* RNAi had no effect. Bottom bands indicate MH33 [90] levels in each lane as a loading control. Quantification (Table S1) using ImageJ software revealed that CKI-1::GFP level was increased by RNAi of *pat-3*, *ina-1*, *unc-97*, *unc-52, pat-6,* and *let-2*. Panel C: CKI-1::GFP expression levels were also measured in *pat-3*(+) animals treated with E3 ligase gene RNAi. Top bands represent the amount of CKI-1::GFP in the extracts prepared from each RNAi condition. L4440 is a negative RNAi control. The *skpt-1*, *lin-23* and *cul-1* RNAi depletions caused upregulation of CKI-1::GFP, while *rbx-1* and *cul-4* RNAi had no effect. Bottom bands indicate MH33 [90] levels in each lane as a loading control. Quantification (Table S1) using ImageJ software revealed that the CKI-1::GFP level was increased by RNAi of *skpt-1*, *lin-23* and *cul-1*.

In the *pat-3(sp)* rescued animals, CKI-1::GFP localization was visibly different from that seen in *pat-3(+)* animals. In contrast to the compact, nucleolar staining seen in *pat-3(+)* animals, CKI-1::GFP in *pat-3(sp)* was clumped and accumulated in a ring around a dark center in the nucleus (Figures 2B and 2D), suggesting mislocalization and possible exclusion from the nucleolus. In addition, the intensity of green fluorescence in *pat-3(sp)* was increased compared to *pat-3(+)*. In order to test for a possible correlation between the level of CKI-1::GFP and the integrin (*pat-3(+)* or *pat-3(sp)*) expressed, we first analyzed the amount of CKI-1::GFP in the *pat-3* rescued lines. Protein lysates were prepared from an equal number of L4/young adult transgenic animals and tested for CKI-1::GFP protein levels. CKI-1::GFP level in *pat-3(sp)* was ten fold more intense than that seen in *pat-3(+)* lysates (Figure 3A), suggesting that PAT-3 signaling may control CKI-1 levels.

**Figure 4:**
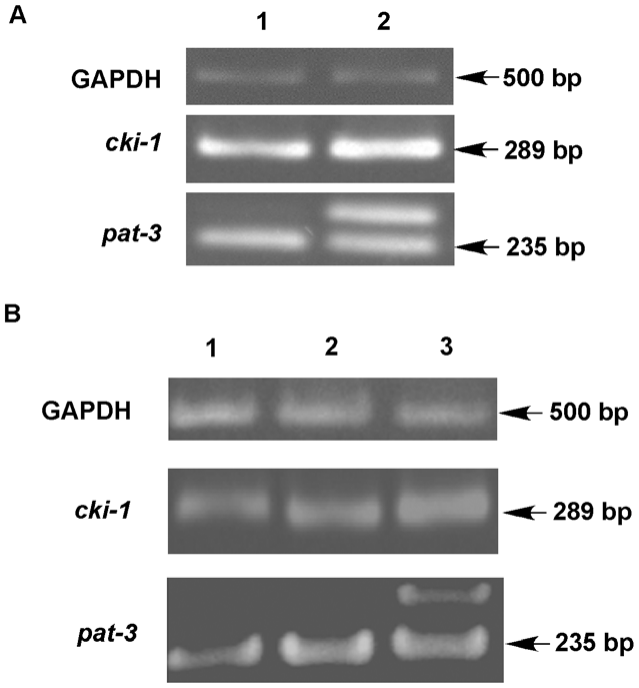
RT-PCR analysis of *cki-1* mRNA in *cki-1*::GFP transgenic and β*pat-3* rescued lines lacking *cki-1*::GFP. Panel A: Lanes 1 and 2 display the level of mRNA in *pat-3(+)* and *pat-3*(*sp*) animals, respectively. The GAPDH (glyceraldehyde-3-phosphate dehydrogenase) gene, *gpd-1*, was used as an mRNA loading control. The level of each transcript was measured using ImageJ software. Quantification of cDNA using ImageJ software revealed that *cki-1* mRNA levels were similar in all lines compared (Table S1). Panel B: *cki-1* mRNA was measured in β*pat-3* rescued lines lacking the CKI-1::GFP construct. Lanes 1–3 are N2, JE443 *pat-3(+),* and BU7221 *pat-3(sp)*. The GAPDH gene, *gpd-1*, was used as an mRNA loading control. Quantification of cDNA levels using ImageJ software revealed that *cki-1* mRNA levels were similar in all lines compared (Table S1).

Because the immunoblot results revealed that *pat-3(sp)* animals produced more CKI-1::GFP protein than *pat-3(+)*, we next assessed the effect on *cki-1* transcription. RNA from each rescued line was isolated and analyzed for the amount of *pat-3* or *cki-1* mRNA using RT-PCR (Figure 4A). We also measured the *cki-1* mRNA level in BU7221, a *pat-3(sp)* rescued line without *cki-1*::GFP [34]. No significant differences were seen in any of the experiments, suggesting that *pat-3(sp)* does not significantly increase the level of *cki-1* mRNA compared to controls (Figure 4B).

### Integrin Signaling Regulates CKI-1 Localization

In order to define genetic pathways that link PAT-3 integrin to CKI-1, a series of RNAi experiments were performed. We hypothesized that the integrin effect on the localization of CKI-1::GFP in *pat-3(sp)* would be mediated by genes that interact with the cytoplasmic domain of β integrin. Thus, candidate genes were selected from known focal adhesion components [44]. Previous analysis of embryonic muscle development identified 20 essential genes, mostly encoding components of dense bodies and M-lines, which are analogous to focal adhesions [44]–[45]. Integrins are located in the base of these structures and anchor the sarcomeres to the basement membrane. Data from the SAGE database [37] indicated ten of the integrin signaling genes were expressed in the hypodermis. To screen for the genes involved in CKI-1 localization, we tested if RNAi depletion of these integrin signaling components [44] in *pat-3(+)* would result in mislocalization of CKI-1::GFP in the nucleoplasm, similar to that seen in *pat-3(sp)* animals (Results summarized in Table 1).

**Table 1:** RNAi analysis of genes involved in localization pattern.

Tested Gene	*pat-3(+)*	*pat-3(sp)*	*SAGE search in hypodermis* *
*pat-3/β* integrin	++ (80)	+++ (80)	Positive
*ina-1/α* integrin	++ (80)	+++ (50)	Positive
*pat-4/*ILK	++ (35)	+++ (35)	Positive
*unc-97/*PINCH	+++ (80)	+++ (80)	Positive
*pat-6/*parvin	0 (60)	+++ (60)	Positive
*unc-52/*perlecan	++ (50)	+++ (45)	Positive
*epi-1/*laminin α	0 (15)	+++ (15)	Positive
*let-2/*collagen IV	0 (20)	+++ (20)	Positive
*unc-112/*kindlin	0 (40)	+++ (40)	Positive
Y71G12B.11*/talin*	0 (35)	+++ (35)	Positive
*lin-23/*F-Box	++ (60)	+++ (60)	Positive
*cul-1/*CUL1	++ (80)	+++ (70)	Positive
*skpt-1/*SKP2	++ (70)	+++ (70)	Positive
*rbx-1/ROC1*	0 (20)	+++ (20)	Positive
*cul-4/CUL4*	0 (30)	+++ (30)	Positive
L4440 (vector)	0 (200)	+++ (200)	N/A

% mislocalization refers to animals with mislocalization out of total animals observed. (n) = the number of animals examined. 0 = 0% mislocalization, + = 1–25% mislocalization, ++ = 26–50% mislocalization, +++ = 51–75% mislocalization.

* Name of the gene was queried individually against the SAGE database.

**Figure 5:**
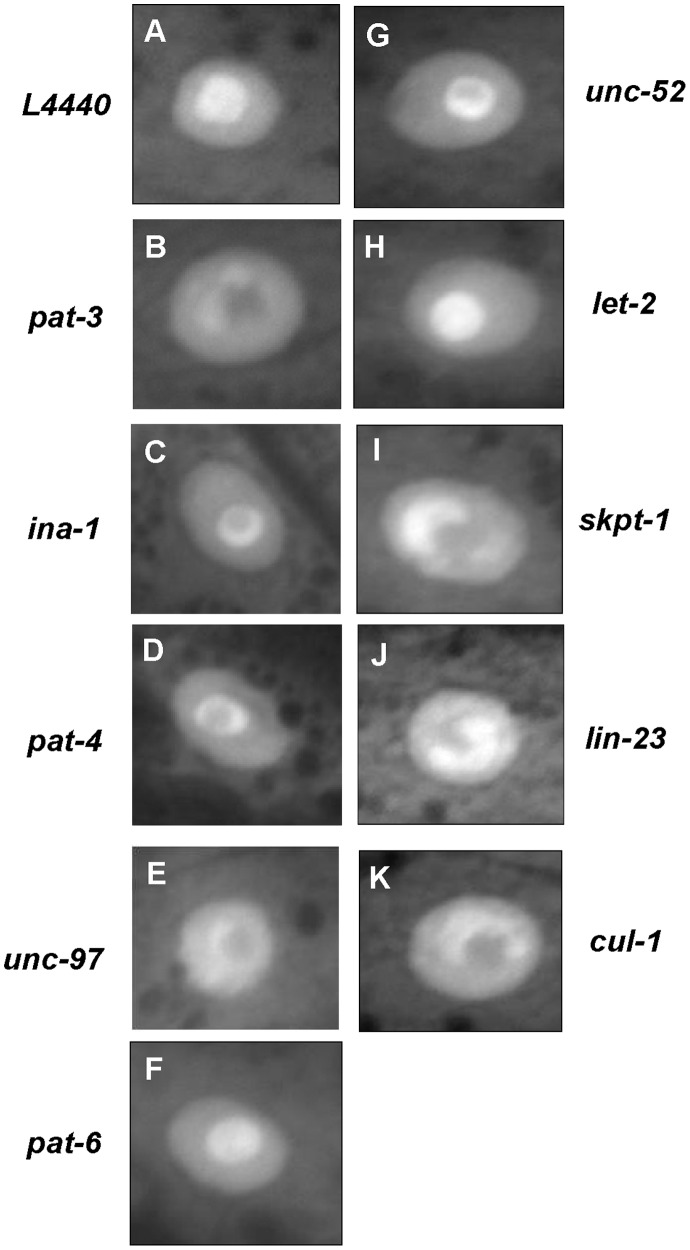
RNAi analysis showed that some focal adhesion and E3 ligase genes are required for CKI-1::GFP localization. Panels depict the results of RNAi of focal adhesion genes, dense body or M-line components, on the localization of CKI-1::GFP. Panels A: the negative control L4440 plasmid, B: *pat-3* RNAi, C: *ina-1* RNAi, D: *pat-4* RNAi, E: *unc-97* RNAi, F: *pat-6* RNAi, G: *unc-52* RNAi, H: *let-2*, I: *skpt-1* RNAi, J: *lin-23* RNAi and K: *cul-1* RNAi.

In *pat-3(+)* animals, *pat-3(RNAi)* resulted in CKI-1::GFP accumulation in the nucleoplasm similar to that seen in *pat-3(sp)* (Figures 5B and 2D). Next, integrin α subunits were depleted. Depletion of *ina-1* in the *pat-3(+)* animals also resulted in abnormally clumped CKI-1::GFP (Figures 5C), suggesting that the CKI-1 localization is integrin dependent. Among the focal adhesion genes, *pat-4*/ILK [46], *unc-97*/PINCH [47] and *pat-6*/parvin [48] together form an IPP complex, which is implicated in the control of signaling pathways by the phosphorylation of downstream targets [49]. RNAi of *pat-4*/ILK or *unc-97*/PINCH in *pat-3(+)* resulted in the expected uncoordinated phenotypes (Figure S2) and CKI-1 mislocalization in hypodermal nuclei (Figure 5D and 5E). However, in *pat-6*/parvin RNAi animals, CKI-1 maintained its wild-type localization, possibly suggesting that the CKI-1 localization is independent of parvin (Figure 5F). Because *pat-6* RNAi did not result in a strong uncoordinated phenotype (Figure S2), it is possible that the *pat-6* RNAi is not as effective as the RNAi to *pat-4* and *unc-97*. However, our data is consistent with the interpretation that ILK and PINCH are mediating integrin signals to control CKI-1 localization in the nucleus.

Our RNAi screen also found that *unc-52*/perlecan, a basement membrane component and presumptive integrin ligand [50]–[52], is required for the proper localization of CKI-1. RNAi of *unc-52* in *pat-3(+)* affected the CKI-1 localization pattern (Figure 5G). In contrast, depletion of other basement membrane components, such as *let-2*/collagen IV [53], failed to affect the localization (Figure 5H), suggesting that a subset of ECM components is required for CKI-1 localization.

### Ubiquitin-mediated Protein Degradation Regulates Localization of CKI-1::GFP

Next, we investigated the mechanism by which integrin regulates CKI-1 protein levels without affecting RNA levels. One plausible explanation is that integrin signaling leads to the degradation of CKI-1 [23]. Integrin-triggered p27^KIP1^ degradation has been observed in mammalian cells. For example, integrin crosstalk with receptor tyrosine kinase (RTK) induces the production of SCF^SKP2^ [54], a member of the SCF E3 ubiquitin ligase complex, which binds to the SKP1 [55], CUL1 [56] and FBX-1 [23] E3 ligase complex. This SCF complex targets CDK/cyclin inhibitors such as p27^KIP1^ and p21^CIP1^ [57].

We hypothesized that the SCF complex might play a similar role in the localization and level of CKI-1 in response to integrin signals. To test this hypothesis, we performed RNAi analysis of *skpt-1*/SKP2 [33], *cul-1*/CUL1 [58] and *lin-23*/F-Box [59] and monitored CKI-1 localization (Figure 5I, 5J, and 5K). We first examined *skpt-1/*SKP2 (RNAi) in the *pat-3(+)* background. CKI-1::GFP accumulation in the *pat-3(+)*; *skpt-1* (RNAi) nucleoplasm was almost identical to that seen in *pat-3(sp)* animals (Figure 5I). Similar results were obtained in *cul-1* and *lin-23* RNAi in the *pat-3(+)* strain (Figure 5J and 5K), suggesting ubiquitin-mediated protein degradation is responsible for the proper localization of CKI-1. RNAi of another E3 ligase complex gene, *rbx-1/ROC1* [23], did not alter the localization pattern of CKI-1 (Table 1). Studies have also suggested that the DDB-1/CUL-4 associated factors (DCAF) complex is responsible for p27 degradation in mammals and *Drosophila* [60]. Our analysis of *cul-4(RNAi)* treated *pat-3(+)* animals suggests that this complex is unlikely to be involved in CKI-1 localization in *C. elegans* (Table 1). Taken together, our RNAi analyses suggested that the members of SCF complex play a role in the CKI-1 localization.

### Immunoblot Analysis Demonstrates a Correlation between CKI-1 Overexpression and Mislocalization

We next investigated whether depletion of the focal adhesion and SCF complex genes would affect expression levels of CKI-1/p27^KIP1^ in addition to affecting nuclear localization patterns (Figures 5; Table 1). Immunoblot analyses of CKI-1::GFP were performed using protein extracts from RNAi-treated adult animals (Figure 5A and 5B). The amount of CKI-1::GFP generally increased in protein extracts of focal adhesion and SCF complex RNAi treated animals. Depletion of focal adhesion genes such as *pat-3, ina-1, unc-97, unc-52, pat-6* and *let-2* resulted in an up to 3-fold increase in CKI-1::GFP levels (Figure 3B, Table S1). RNAi of SCF E3 ligase genes such as *skpt-1, lin-23,* and *cul-1* also produced significant increases in CKI-1::GFP levels. (Figure 3C, Table S1). Interestingly, even though nucleolar localization was not affected by *pat-6* and *let-2* RNAi, protein levels were significantly increased (Figure 3B). Our analysis suggested that disruption of integrin signaling or SCF–mediated protein degradation can result in the mislocalization as well as increased expression of CKI-1::GFP. However, the *let-2* and *pat-6* RNAi results suggest there may not be a direct relationship between protein levels and localization.

## Discussion

In this study, integrin regulation of CKI-1 was assessed *in vivo*. Our analysis revealed that CKI-1/p27^KIP1^ had an abnormal localization pattern in the nucleoplasm of animals expressing a mutant integrin, *pat-3(sp)*. In the mutant animals, CKI-1::GFP was overexpressed and clumped in the nucleoplasm, while in animals expressing wild type integrin, CKI-1::GFP was localized predominantly to the nucleolus (Figure 2). Further studies revealed that the amount of CKI-1::GFP protein was increased in the *pat-3* mutant. To delineate the genetic pathway responsible for the upregulation of CKI-1/p27^KIP1^, we depleted focal adhesion and SCF E3 ubiquitin ligase genes in *pat-3(+)* animals and found that these genes are essential for the proper localization and expression of CKI-1::GFP. We conclude that the inhibition of integrin signaling and protein degradation significantly affects CKI-1 protein localization and expression level *in vivo*.

Previous studies have suggested a link between the mammalian integrin splice variant β1C and p27^KIP1^. For example, increased expression of β1C integrin elevated p27^KIP1^ in prostate cancer cell lines [13], [21], [61]. Our *pat-3(sp)* mutant splice form is an artificial variant [34], however, our study found that *pat-3(sp)* behaves similarly to the β1C variant of mammalian β1. *pat-3(sp)* increases CKI-1/p27^KIP1^ expression, possibly leading to a cell cycle arrest.

### CKI-1 is Localized to the Nucleolus

Our observations of nuclear morphology and experiments using *ncl-1* (RNAi) strongly suggest a nucleolar localization for CKI-1::GFP in *pat-3(+)*. In contrast, CKI-1::GFP appeared clumped and was irregularly distributed in the nucleoplasm of *pat-3(sp)* animals, suggesting ectopic accumulation of CKI-1. This nucleolar to nuclear transition in CKI-1::GFP localization suggests a possible link between CKI-1 and nucleolar function.

The nucleolus is the main site of ribosome biogenesis and ribosomal RNA (rRNA) synthesis [62]. The rRNAs are synthesized and assembled into a ribosomal complex in the nucleolus. In mammals, stressed ribosomal synthesis leads to cell cycle arrest via increased p27^KIP1^ levels [63]. In addition, mutations in the cytoplasmic tail of β4 integrin or p27 β4 binding protein (p27^BBP/eIF6^) result in an inability of β4 to localize to hemidesmosomes and a defect in assembly of the 80S ribosomal subunit, suggesting a connection between integrin signaling and ribosome biogenesis [64]–[66]. Although further studies are required, we speculate that the splice defective *pat-3(sp)* integrin may cause a decrease in ribosome biosynthesis.

### Proper CKI-1 Localization is Linked to Integrin and Integrin-associated Molecules

Integrins are αβ heterodimers and both subunits are necessary for integrin function. Therefore, depletion of the α subunit would have the same effect as disruption of the β subunit, PAT-3. Indeed, CKI-1::GFP mislocalization and increased expression were observed when the α integrin gene *ina-1* was depleted by RNAi, suggesting that CKI-1 upregulation is a result of disrupted integrin function and that integrin activity is normally required for proper CKI-1 localization and protein levels. In addition, RNAi analysis of the IPP complex showed that *pat-4*/ILK [46], [67], *unc-97*/PINCH [47] and *pat-6*/parvin [48] are also required for correct expression and/or localization of CKI-1. Some studies have suggested that these components act together and are degraded if *pat-4*/ILK or *unc-97*/PINCH is not present [68] but other studies, including ours, suggest these molecules may have independent roles [69], [70]. In addition, PINCH or parvin binding to ILK is mutually exclusive, suggesting that PINCH might provide a different mode of signaling than parvin [71], [72]. ILK has been shown to play an important role in cell proliferation in tissue culture [73] and PINCH is frequently upregulated in human cancers [74]. Disruption of ILK or PINCH inhibits cell proliferation and increases the expression level of p27^KIP1^ and pRb [75], suggesting that cells might generally respond to perturbed cell adhesion by inducing p27^KIP1^ expression and halting the cell cycle. In addition, our analysis links the ECM ligand, *unc-52*/perlecan, to CKI-1 localization, suggesting that perlecan-bound integrin might regulate CKI-1 by activating *pat-4*/ILK and *unc-97*/PINCH.

### SCF E3 Ligase Complex is Required for the Proper Localization of CKI-1

This study revealed that ubiquitin-mediated protein degradation plays a crucial role in regulating CKI-1/p27^KIP1^. RNAi of SCF E3 ligase genes resulted in mislocalization of CKI-1::GFP. The E3 ligase includes a scaffold protein (CUL-1/CUL1) that assembles the ubiquitin ligation complex along with ubiquitin transferase (RBX-1/ROC1), an adaptor (SKPT-1/SKP2), and a substrate-binding protein (LIN-23/F-Box). The SKP2/Cullins/F-box (SCF) E3 ligase complex is involved in the degradation of cellular proteins such as cell cycle inhibitors, transcription factors and other signaling effectors [76]. It has been reported that phosphorylated p27^KIP1^ is degraded in an SCF-dependent manner and that the loss of SCF is associated with pathological conditions such as cancer [76]–[78]. Our RNAi analyses showed that SKPT-1/SKP2 [33], [55], LIN-23/F-Box [23], [59] and CUL-1/CUL1 [56], [58], [77] are required for the proper localization of CKI-1, while RBX-1/ROC1 showed no effect. This might indicate that SCF ligase forms a complex with a protein other than RBX-1 for the degradation of CKI-1.

### Integrin Signaling May Influence the Cell Cycle by Regulating CKI-1 Level via SCF Ubiquitin Ligases

**Figure 6:**
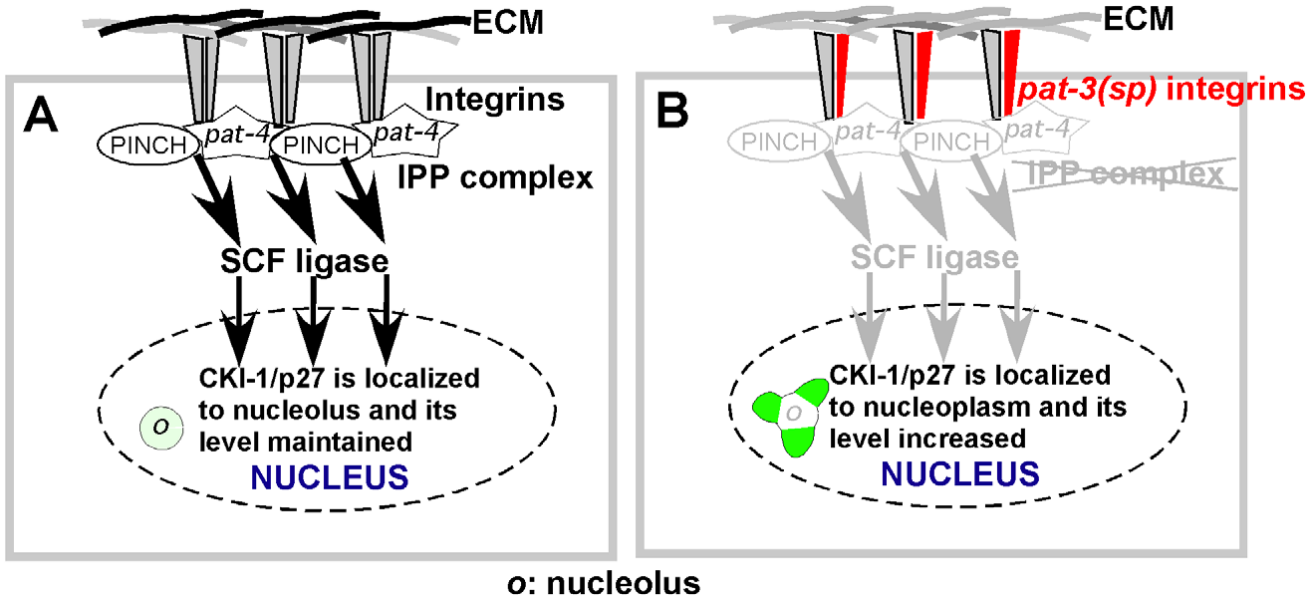
Model for the integrin regulation of CKI-1. When ECM ligand binds to integrin on the cell surface, the IPP complex delivers signals to the SCF E3 ligase complex resulting in sequestration of CKI-1/p27 to the nucleolus. In *pat-3(sp)*, the mutant β integrin interferes with the function of wild-type *pat-3(+)*. Consequently, integrin does not signal to the IPP complex. We suggest the reduced integrin signals may leave the SCF complex inactive. This allows an increase in the amount of CKI-1/p27 and alters the location of the protein in the nucleus.

We propose a potential model for the role of integrin signaling in CKI-1 regulation. Our preferred model assumes the presence of functional integrin in the hypodermal cells. Integrin is activated by binding to ECM ligands and signaling is initiated and propagated by molecules such as *pat-4*/ILK and *unc-97*/PINCH to SCF ligase which degrades CKI-1 (Figure 6). *pat-3(sp)* may interfere with the formation of the SCF complex and the degradation of CKI-1 by inhibiting the function of wild-type PAT-3 integrins or by acting as a non-functional β subunit that significantly dilutes integrin signaling [8]. SAGE analysis indicates that *pat-3* and *ina-1* are expressed in hypodermal cells [79], [80] consistent with a previously identified role for *ina-1/*α integrin function in hypodermis [24]. Although we have identified a role for the SCF complex in CKI-1 degradation, our work does not specifically address whether SCF activation is directly linked to integrin signaling in a linear manner, as displayed in the model (Figure 6). Future genetic studies should determine the cell autonomy and epistatic relationships of the genes in the pathway from integrin to cell cycle control.

In addition to muscle and gonad morphogenesis, our work identifies another important function of integrin in *C. elegans*, the regulation of CKI-1/p27^KIP1^. This finding brings new insight onto cell cycle control. Integrins appear to regulate the level of p27^KIP1^ via signaling mediators such as *pat-4*/ILK and *unc-97*/PINCH and to maintain SCF ubiquitin ligase activity. Importantly, *unc-52* RNAi produces a similar phenotype, suggesting that the cell-matrix interaction balances the amount of CKI-1 in the cell. This information will be useful in understanding the mechanism on how the cell-ECM interaction regulates cell cycle progression.

## Materials and Methods

### Animals and Culture

*Caenorhabditis elegans* were cultured on nematode growth medium (NGM) agar plates seeded with OP50 *E. coli* under standard conditions [81]. RW3600 qC1 *dpy-19 (e1259) glp-1 (q339)/pat-3 (st564)* III [38] were acquired from the *Caenorhabditis* Genetics Center (St. Paul, MN). The *pat-3* transgenic rescued lines used in this study are listed in Table 2. All transgenic lines were cultured under standard conditions [81].

**Table 2:** Transgenic C. elegans used in this study.

Mutant designation	Constructs injected	Reference
*pat-3(+) rab-3::RFP, cki-1::GFP (*BU444 *kqEx75)*	*pat-3(st564)*, pPAT3(+), pGH8 *rab-3*::RFP, pVT352G *cki-1*::GFP	This study
*pat-3(+) myo-3::RFP, cki-1::GFP (*BU445 *kqEx76)*	*pat-3(st564)*, pPAT3(+), pCFJ104 *myo-3*::RFP, pVT352G *cki-1*::GFP	This study
*pat-3(+) rab-3::RFP (BU446 kqEx77)*	*pat-3(st564)*, pPAT3(+), pGH8 *rab-3*::RFP	This study
*pat-3(+) sur-5::GFP (JE443)*	*pat-3(st564)*, pPAT3(+), TG96 *sur-5*::GFP	[84]
*pat-3(sp rab-3::RFP, cki-1::GFP (*BU7222 *kqEx73)*	*pat-3(st564)*, pPAT3-sp, pGH8 *rab-3::*RFP, pVT352G *cki-1::*GFP	This study
*pat-3(sp) myo-3::RFP, cki-1::GFP (*BU7223 *kqEx74)*	*pat-3(st564)*, pPAT3-sp, pCFJ104 *myo-3::*RFP, pVT352G *cki-1::*GFP	This study
*pat-3(sp) sur-5::GFP (*BU7221 *kqEx21)*	*pat-3(st564)*, pPAT3-sp, TG96 *sur-5*::GFP	[34]

Multiple transgenic lines of each β*pat-3* rescue were analyzed.

### Mutant Pat-3 Constructs and Germline Tranformation

pPAT3 (+)-PB12K and mutant constructs for pPAT3-sp were created using overlap extension PCR and have been described previously [34]. Germline transformation was performed using the standard protocol for microinjections [82]. Briefly, pPAT3 constructs were mixed with *cki-1::GFP* [26] and *rab-3::RFP* or *myo-3::RFP* [83]. All rescued lines were made at a mixture of 5 µg/ml of pPAT3, 2 µg/ml of pVT352G (*cki-1*::GFP), and 100 µg/ml of pGH8 (*rab-3::RFP)* or 100 µg/ml of pCFJ104 (*myo-3*::RFP) in TE buffer (pH 7.5). This mixture was injected into a distal gonad of the RW3600 qC1 *dpy-19(e1259) glp-1(q339)/pat-3(st564)* III animal. F2 generation animals with 100% red progeny were isolated and more than 10 generations elapsed before the characterization of phenotypes and multiple lines were used for confirmation [84].

### Phenotype Characterization and Nuclei Expression Pattern Identification

To characterize rescued lines, young adult worms were mounted in a drop of M9 buffer containing 1% NaN_3_ (Sigma Chemical Co., St. Louis, MO) or 0.5 mM levamisole on a 24×60 mm coverslip coated with 4% agarose and examined on a Nikon TE2000-U Diaphot epifluorescence microscope. Images were captured using a CoolSnap *cf* monochrome camera (Roper Scientific, Tucson, AZ) and analyzed with Metavue imaging software (version 7.5, Molecular Devices Co., Downingtown, PA). Typically, CKI-1::GFP became visible at the late L4 stage. In the wild-type rescue *pat-3*(+) animals, GFP was apparently nucleolar: a strong green spot on a larger green nucleus. To photograph the image, the camera was set at the exposure time of 2000 milliseconds. However, in *pat-3(sp)* animals, the CKI-1::GFP expression was much brighter than in the *pat-3*(+). For *pat-3(sp),* images were taken at the exposure time of 300 milliseconds. About 20 hypodermal nuclei in the midbody, an area including vulva at the ventral midline, of each animal was observed for nuclear morphology characterization. Displayed images are patterns seen most commonly under the described conditions.

To analyze the size of nucleoli, the area was measured using ImageJ software (version 1.33, National Institute of Health, Rockville, MD) [85]. To determine the nucleolar to nuclear ratio, the area value of nucleolus, a brighter spot on a nucleus, was divided by that of nucleus. Five measurements for each rescued line were averaged for comparison.

### Reverse Transcription Polymerase Chain Reaction (RT-PCR)

To analyze *cki-1*, *pat-3*, and *gpd-1*/GAPDH mRNA levels, animals were partially synchronized by isolating embryos using 20% alkaline hypochlorite solution [86]. After 48 hours, forty L4 to young adult animals were picked into 10 µL of M9 buffer. RNA was extracted using 250 µL Tri-Reagent (Sigma-Aldrich, St. Louis, MO) and 50 µL chloroform (1/5 volume of Tri-Reagent) and RNA was precipitated from the extract with isopropanol. Approximately 1 µg of total RNA was used to synthesize cDNA with Transcriptor Reverse Transcription Kit (Roche, Carlsbad, CA) primed with random hexamers in a total 20 µL reaction volume. Total of 1 µL cDNA was used in PCR amplification with *cki-1* primers, *pat-3* primers, and with control *gpd-1*/GAPDH primers previously described [34]. Primer sequences listed below were used for amplification:

CKI1 Forward 2: 5′-GGAGTTCTACAGAACC-3′

CKI1 Reverse 2: 5′-CACCGGAGACAGCTTG-3′

PAT3PT Forward1: 5′-CTCAACGAAACTACACCCTGCC-3′

PAT3PT Reverse 1: 5′-TTAGTTGGCTTTTCCAGCGTATACTGG-3′.

### Immunoblot Analysis

For quantitative analysis of CKI-1 protein levels in each strain, we first picked 30 young adults into 10 µL of M9 buffer and 10 µL of 2X Laemmli Sample Buffer (Bio-Rad Laboratories, Hercules, CA) premixed with 1∶1000 β-mercaptoethanol. Sample solutions were then boiled for 10 minutes at 100°C and electrophoresed through a 10% SDS-polyacrylamide gel at 180 V for 1 hour. Isolated proteins bands were electrotransferred onto a nitrocellulose membrane (Whatman Ltd., Dassel, Germany) using wet transfer at 100 V for 75 minutes in BSN transfer buffer with no methanol or SDS. This nitrocellulose membrane was then blocked for 1 hour in 5% milk solution at room temperature. Rabbit polyclonal IgG anti-GFP antibody (ab290, Abcam Inc., Cambridge, MA) at 1∶2000 was applied overnight at 4°C as primary antibody and goat anti-rabbit IgG HRP conjugated (ab6721, Abcam Inc., Cambridge, MA) at 1∶5000 was applied for 1 hour at RT. For control blots, LS25, a monoclonal antibody against UNC-54, or MH33, a monoclonal antibody against a gut specific intermediate filament protein, were diluted at 1∶1000 and 1∶2000. The primary antibody solutions were applied and detected by the goat anti-mouse IgG HRP conjugated (Sigma Chemical, Mo) secondary antibody. ECL chemiluminescence reagents (Thermo Fisher Scientific, Rockford, IL) were added to the membrane for 1 minute before exposure to the ULTRA-LUM gel imager (Ultra-Lum Inc., Claremont, CA) and analyzed with UltraQuant software (Ultra-Lum Inc., Claremont, CA). Individual band intensity was quantified using ImageJ software (version 1.66, National Institute of Health, Rockville, MD) that measured the integrated density of each band to analyze the intensity of bands.

### RNA-mediated Interference of Gene Expression (RNAi) Analysis

*C. elegans* RNA interference analysis was performed using the bacterial feeding method [87], [88]. In addition to the standard RNAi protocol, we synchronized the stage of animals; embryos were collected using the standard 20% alkaline hypochlorite solution method [86]. After washes, collected embryos were placed onto RNAi plates, which were incubated in 20°C for 3 to 4 days until young adulthood before characterization. About 20 hypodermal nuclei in the midbody of transgenic worms were observed for the characterization of nuclear morphology using Metavue software. To verify the efficiency, all RNAi animals were examined for behavioral phenotypes linked to the RNAi gene. For example, focal adhesion gene RNAi was subjected to thrashing assays [89] because uncoordinated (Unc) phenotypes were previously reported for RNAi of these genes (Figure S2). Each RNAi-inducing plasmid (Geneservice, Hinxton, UK) used in this study was isolated and sequenced in order to verify the gene targeted by the construct.

## Supporting Information

Figure S1 ***ncl-1(RNAi)*** **increases the size of the nucleolus in CKI-1::GFP in** ***pat-3*** **transgenic animals.** Panel A: *ncl-1(RNAi); pat-3(+).* The area of CKI-1::GFP is 2.4 times (P<.001) the size of the area seen in the no RNAi control in panel B: *CKI-1::GFP* in *pat-3(+)* background.
(TIF)

Figure S2 **Locomotion defects of RNAi animal.** Panel A: Number of body bends in 30 seconds was measured in *pat-3(+)* animals treated with RNAi of *pat-3, ina-1, unc-97, unc-52, pat-6, let-2,* and *unc-112* genes. The number of body bends was compared to that of L4440, the negative control RNAi. Black bars indicate the average number of body bends for each RNAi tested. Horizontal bars indicate the standard error of each test. N = 10. *indicates P<.0001 (compared to L4440). Panel B: Number of body bends in 30 seconds was measured in *pat-3(+)* animals treated with RNAi of *rbx-1, cul-4, skpt-1, lin-23,* and *cul-1* genes. The number of body bends was compared to that of L4440, the negative control RNAi. Bars indicate the average number of body bends for each RNAi tested. Horizontal bars indicate the standard error of each test. N = 10. *indicates P<.0001 (compared to L4440).
(TIF)

Table S1 **ImageJ analysis data.**
(XLSX)
