# Supplementary figures and images for: A Novel Mutation in β Integrin Reveals an Integrin-Mediated Interaction between the Extracellular Matrix and cki-1/p27KIP1
Source: PLoS One. 2012 Aug 6;7(8):e42425. doi: 10.1371/journal.pone.0042425 (PMC3412830; doi:10.1371/journal.pone.0042425)

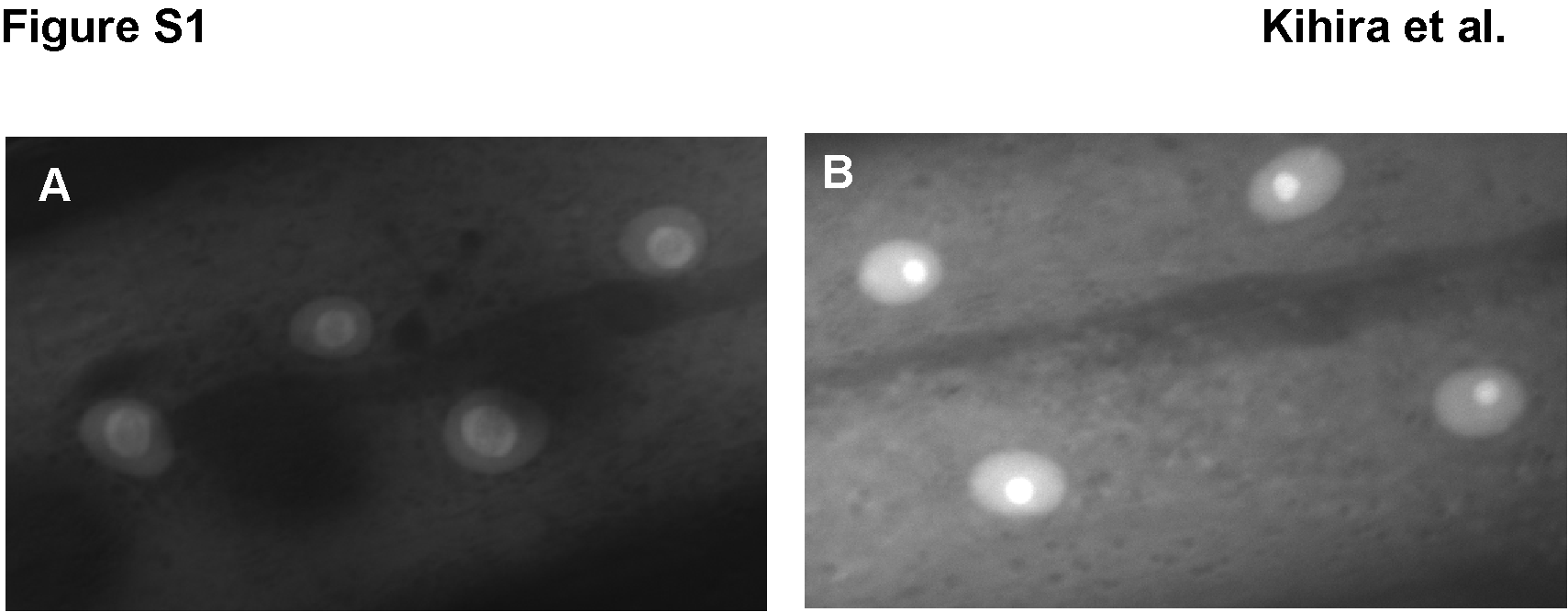

Supplement: Figure S1 — ncl-1(RNAi) increases the size of the nucleolus in CKI-1::GFP in pat-3 transgenic animals. Panel A: ncl-1(RNAi); pat-3(+). The area of CKI-1::GFP is 2.4 times (P<.001) the size of the area seen in the no RNAi control in panel B: CKI-1::GFP in pat-3(+) background. (TIF) [file pone.0042425.s001.tif]

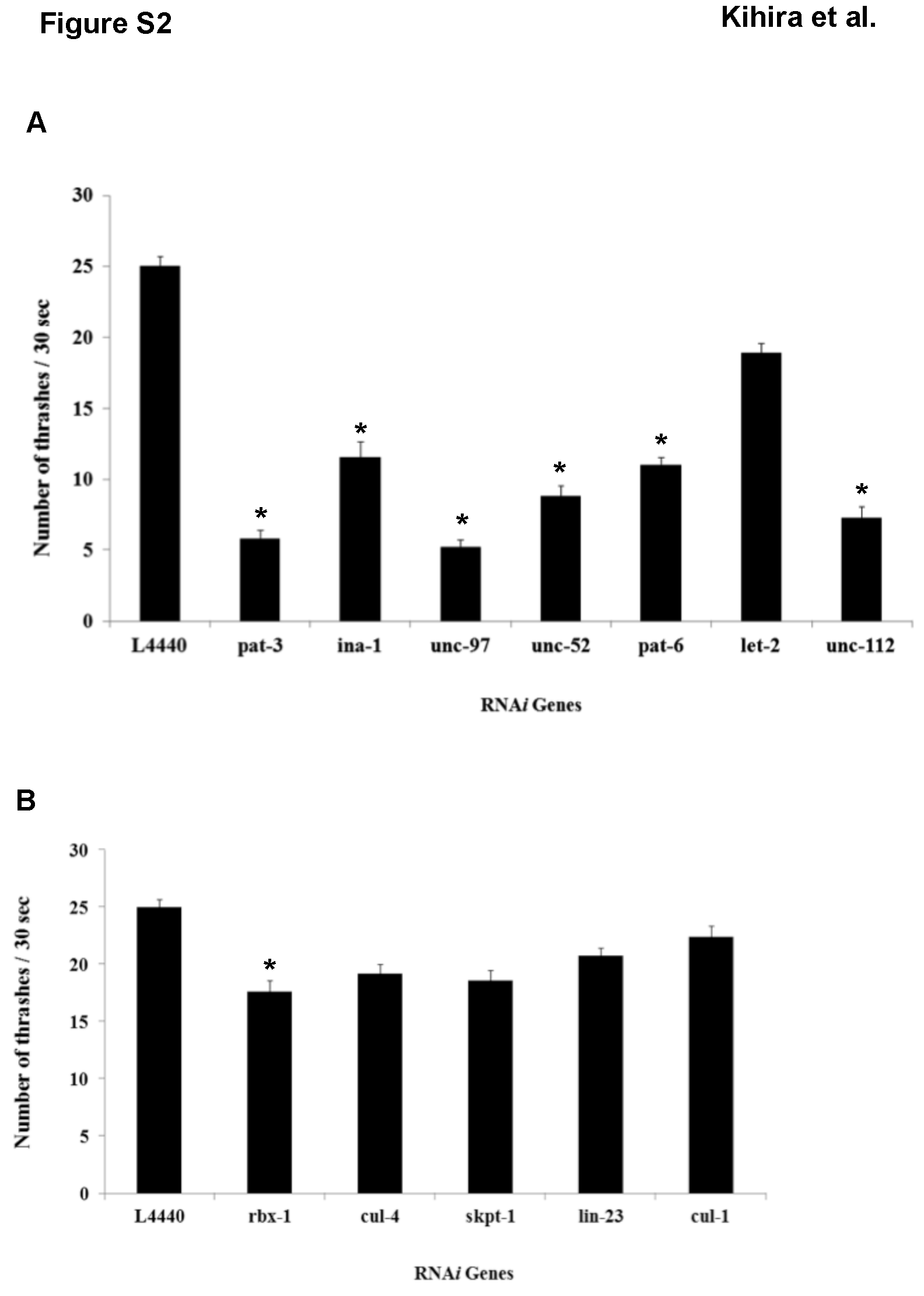

Supplement: Figure S2 — Locomotion defects of RNAi animal. Panel A: Number of body bends in 30 seconds was measured in pat-3(+) animals treated with RNAi of pat-3, ina-1, unc-97, unc-52, pat-6, let-2, and unc-112 genes. The number of body bends was compared to that of L4440, the negative control RNAi. Black bars indicate the average number of body bends for each RNAi tested. Horizontal bars indicate the standard error of each test. N = 10. *indicates P<.0001 (compared to L4440). Panel B: Number of body bends in 30 seconds was measured in pat-3(+) animals treated with RNAi of rbx-1, cul-4, skpt-1, lin-23, and cul-1 genes. The number of body bends was compared to that of L4440, the negative control RNAi. Bars indicate the average number of body bends for each RNAi tested. Horizontal bars indicate the standard error of each test. N = 10. *indicates P<.0001 (compared to L4440). (TIF) [file pone.0042425.s002.tif]
